# Necroptosis contributes to deoxynivalenol-induced liver injury and inflammation in weaned piglets

**DOI:** 10.1186/s40104-024-01117-1

**Published:** 2024-12-03

**Authors:** Qilong Xu, Hanqiu Gong, Mohan Zhou, Junjie Guo, Shaokui Chen, Kan Xiao, Yulan Liu

**Affiliations:** Hubei Key Laboratory of Animal Nutrition and Feed Science, Wuhan Polytechnic University, Wuhan, 430023 China

**Keywords:** Deoxynivalenol, Liver damage, Necroptosis, Necrostatin-1, Pigs

## Abstract

**Background:**

The aim of this study was to investigate the role of necroptosis in deoxynivalenol (DON)-induced liver injury and inflammation in weaned piglets.

**Methods:**

In Exp. 1, 12 weaned piglets were divided into 2 groups including pigs fed basal diet and pigs fed diet contaminated with 4 mg/kg DON for 21 d. In Exp. 2, 12 weaned piglets were divided into 2 groups including control piglets and piglets given a gavage of 2 mg/kg body weight (BW) DON. In Exp. 3, 24 weaned piglets were used in a 2 × 2 factorial design and the main factors including necrostatin-1 (Nec-1) (DMSO or 0.5 mg/kg BW Nec-1) and DON challenge (saline or 2 mg/kg BW DON gavage). On 21 d in Exp. 1, or at 6 h post DON gavage in Exp. 2 and 3, pigs were killed for blood samples and liver tissues. Liver histology, blood biochemical indicators, and liver inflammation and necroptosis signals were tested.

**Results:**

Dietary or oral gavage with DON caused liver morphological damage in piglets. Dietary DON led to hepatocyte damage indicated by increased aspartate transaminase (AST) activity and AST/alanine aminotransferase (ALT) ratio, and DON gavage also caused hepatocyte damage and cholestasis indicated by increased AST and alkaline phosphatase (AKP) activities. Dietary DON caused liver necroptosis indicated by increased protein abundance of total receptor interacting protein kinase 3 (t-RIP3) and total mixed lineage kinase domain-like protein (t-MLKL). Moreover, DON gavage increased mRNA expression of interleukin *(IL)-6* and *IL-1β* in liver. DON gavage also induced liver necroptosis demonstrated by increased protein abundance of t-RIP3, phosphorylated-RIP3 (p-RIP3), t-MLKL and p-MLKL. However, pretreatment with Nec-1, a specific inhibitor of necroptosis, inhibited liver necroptosis indicated by decreased protein expression of t-RIP3, p-RIP3, t-MLKL and p-MLKL. Nec-1 pretreatment reduced liver morphological damage after DON gavage. Pretreatment with Nec-1 also attenuated liver damage induced by DON indicated by decreased activities of AST and AKP. Furthermore, Nec-1 pretreatment inhibited liver mRNA expression of *IL-6* and *IL-1β* after DON challenge.

**Conclusions:**

Our data demonstrate for the first time that necroptosis contributes to DON-induced liver injury and inflammation in piglets.

**Supplementary Information:**

The online version contains supplementary material available at 10.1186/s40104-024-01117-1.

## Introduction

Deoxynivalenol (DON) is the most common mycotoxin in cereal products, which was mainly produced by *Fusarium* *graminearum* and *F.* *culmorum* [1]. It is well known that this mycotoxin has a wide range of toxicity [2], and has harmful effects on the health of livestock [3]. Different animals have different sensitivity to its exposure, among which pigs are the most sensitive to it [4]. DON contamination causes diarrhea, anorexia, vomiting, and further growth retardation and other symptoms in pigs [5]. Emerging evidence showed that both low or high concentrations of DON could cause damage of tissues including intestine, liver and kidney in animals [1, 6]. Especially, liver is an important organ for DON-induced toxicity [7]. DON can destroy liver morphology, induce inflammatory response, and increase hepatocyte death [8]. However, the molecular mechanism by which this mycotoxin induces liver injury and inflammation in pigs remains unclear.

Liver injury is closely related to hepatocyte death. There are several types of cell death, including necrosis, apoptosis and autophagy [9]. Traditionally, cell necrosis was thought to be an uncontrolled type of cell death [10, 11]. In recent years, a new cell death mode, necroptosis, has been identified and attracted researcher’s attention because it is different from other cell death [12]. Necroptosis is an active and orderly way of cell death determined by genes, independent of the cysteine aspartate protease (caspase) pathway, and generally occurs when apoptosis is inhibited, and eventually triggers inflammatory response of neighboring cells [13]. Necroptosis morphologically exhibits the features of necrosis [14]. However, it is regulated by a unique signaling pathway involving the key regulators such as receptor interacting protein kinase 1 (RIP1), RIP3, and mixed lineage kinase domain-like protein (MLKL) [15]. Previous research has shown that necroptosis plays an important role in tissue damage caused by multiple factors such as ischemia-reperfusion and inflammatory response [16–18]. Additionally, inhibition of necroptosis signaling pathway could attenuate tissue damage induced by these factors [18–20]. However, it is still unknown whether necroptosis is involved in liver injury and inflammation after DON exposure in piglets.

So far, the role of necroptosis in liver injury caused by DON has not been reported. Therefore, in this study, we firstly fed weaned piglets with DON-contaminated diet to induce chronic live damage. Secondly, piglets were given an oral gavage with DON to induce acute liver damage. Furthermore, a necroptosis inhibitor necrostatin-1 (Nec-1) was used to explore whether necroptosis was involved in DON-induced liver injury and inflammation.

## Materials and methods

### Experimental animals and design

All experiments were approved by the Animal Care and Use Committee of Wuhan Polytechnic University (Wuhan, China). The approval number of the Ethical Committee of Use of Animals was EM20220425001. A total of 48 healthy 28-day-old Duroc × Landrace × Large crossbred weaned piglets with similar weight of 7.1 ± 0.8 kg were purchased from Aodeng Agricultural and Animal Husbandry Technology Co., Ltd. (Hubei, China). In all experiments, pigs were individually penned and there were 6 replicate pens for each treatment. The pigs were adapted for 7 d. DON was cultivated from *Fusarium* *graminearum* W3008 and added to the basal diet [21, 22]. DON for oral gavage was purchased from Qingdao Pribolab Bioengineering Co., Ltd. (Qingdao, China).

In Exp. 1, 12 piglets (7.2 ± 0.2 kg) were randomly divided into 2 groups including control group and DON group. Piglets were fed the control feed or 4 mg/kg DON-contaminated feed for 21 d. The concentration of DON in diet was chosen according to previous research [23, 24] and our preliminary study. The DON concentrations in basal diet and DON-contaminated diet were 0.3 mg/kg and 4 mg/kg, respectively. After 21 d, piglets were weighed and blood samples were collected. Then piglets were anesthetized by intramuscular injection with Zoletil® 50 (10 mg/kg BW) to euthanasia for liver samples. In Exp. 2, another 12 piglets (7.1 ± 0.4 kg) were randomly divided into 2 groups including piglets given a gavage with 2 mg/kg BW DON or an equal volume of normal saline. At 6 h following gavage of saline or DON, blood and liver samples were collected in the same method as Exp. 1. The concentration of DON gavage to piglets was chosen according to previous research [25, 26] and our preliminary study. In Exp. 3, 24 weaned piglets (7.1 ± 0.6 kg) were randomly divided into 4 groups by a 2 × 2 factorial design with 6 pigs in each treatment. Piglets were given a gavage with 2 mg/kg BW DON or an equal volume of normal saline 30 min after pretreatment with intraperitoneal injection of 0.5 mg/kg BW Nec-1 or an equal volume of 5% dimethylsulfoxide (DMSO). Blood samples and liver tissues were collected at 6 h post gavage of DON or saline in the same method as Exp. 1.

### Blood and liver sample collection

Blood samples were collected by jugular puncture into a 10-mL vacuum tube and centrifuged to collect serum. Then serum was stored at −80 °C for hepatocyte damage and cholestasis analysis. After collecting blood samples, piglets were euthanized to gather liver samples (about 0.5 cm^2^). A part of liver samples was immobilized in 4% paraformaldehyde for histological analysis. The other liver tissue was cut into small pieces, quick-frozen in liquid nitrogen and stored at −80 °C for further analysis.

### DON concentration

The concentration of DON in serum and liver was detected by an AgraQuant^®^ DON ELISA Test Kit following the manufacture’s protocol (Romer Labs, Singapore).

### Hepatocyte damage indexes

The activities of alanine aminotransferase (ALT), aspartate aminotransferase (AST) and alkaline phosphatase (AKP) in serum were determined by Hitachi 7020 automatic biochemical analyzer according to the kit instructions (AST: KP100, ALT: KP622, AKP: EK872; Fujifilm Wako Pure Chemical Corp., Tokyo, Japan).

### Liver histology

The liver samples were dehydrated and embedded, then sliced into 5 μm paraffin sections, and finally stained by hematoxylin-eosin (HE) according to He et al. [27]. The liver tissue morphology was observed by Olympus light microscope (Olympus, Tokyo, Japan).

### Liver ultrastructure

Fragments of liver tissue (1.5 mm × 1.5 mm) were fixed in 2.5% glutaraldehyde fixative solution, and the samples were treated and observed by transmission electron microscopy (TEM) in Seville Biotechnology Co., Ltd. (Wuhan, China). The specific methods according to He et al. [27].

### mRNA expression of pro-inflammatory cytokines

Total RNA was extracted from liver tissues using the TRIzol reagent (TaKaRa Biotechnology, Beijing, China) according to the manufacturer’s instruction. After RNA quantitation and reversion, quantitative PCR was carried out on an ABI 7500 Real-Time PCR system (Applied Biosystems, Life Technologies) using a SYBR Premix Ex Taq (Tli RNaseH Plus) qPCR kit (TaKaRa Biotechnology, Dalian, China). Results were analyzed by the 2^−ΔΔCt^ method of Livak and Schmittgen [28], with *GAPDH* as the housekeeping gene. The relative mRNA abundance of each target gene was normalized to the control group. All samples were run in triplicate. Primers used for real-time PCR analyses are listed in Additional file 1.

### Protein expression of necroptosis signals

Liver samples were homogenized by lysis buffer and centrifuged at 4 °C. The supernatants were collected for Western blot and protein assay. After determining the content of the protein, liver proteins were separated on a polyacrylamide gel and transferred onto polyvinylidene difluoride membranes. Immunoblots were blocked with 3% BSA and incubated overnight with primary antibodies. Specific primary antibodies including mouse anti-t-RIP1 (1:1,000, LifeSpan BioSciences, Seattle, Washington, USA), rabbit anti-phosphorylated RIP1 (p-RIP1) (1:2,000, Cell Signaling Technology, Boston, Massachusetts, USA), mouse anti-t-RIP3 (1:1,000, Santa Cruz Biotechnology, Santa Cruz, CA, USA), rabbit anti-phosphorylated RIP3 (p-RIP3) (1:2,000, Cell Signaling Technology, Boston, Massachusetts, USA), rabbit anti-t-MLKL (1:1,000, Cell Signaling Technology, Boston, Massachusetts, USA), rabbit anti-phosphorylated MLKL (p-MLKL) (1:1,000, Cell Signaling Technology, Boston, Massachusetts, USA) and mouse anti-β-actin (1:10,000, Sigma Aldrich, St. Louis, Missouri, USA). Then anti-rabbit IgG HRP-conjugated antibody (1:5,000, AntGene Biotech, Wuhan, China) was incubated at room temperature. After washing, enhanced chemiluminescence ECL kit (Amersham, Piscataway, New Jersey, USA) was used to visualize blots, and the band density was detected and analyzed in Alpha Innotech Imaging System (Syngene, Cambridge, UK). The relative protein abundance of target proteins was expressed as the ratio of target protein/β-actin protein. The phosphorylated proteins were normalized with total protein abundance.

### Statistical analysis

In Exp. 1 and 2, data were analyzed by Statistical Analysis System (SAS, Cary, NC, USA) software for independent-sample *t*-test. In Exp. 3, data were analyzed by ANOVA using the general linear model procedures for a 2 × 2 factorial design. The model included the effects of DON, Nec-1 and their interaction. When significant Nec-1 × DON interactions occurred, multiple comparison tests were performed using Duncan’s multiple comparisons. All data are expressed as mean ± standard error (SE). *P* ≤ 0.05 was considered statistically significant. Instance in which 0.05 < *P* ≤ 0.1 were considered as trends.

## Results

### DON impairs liver morphology and increases liver enzyme activity

**Fig. 1:**
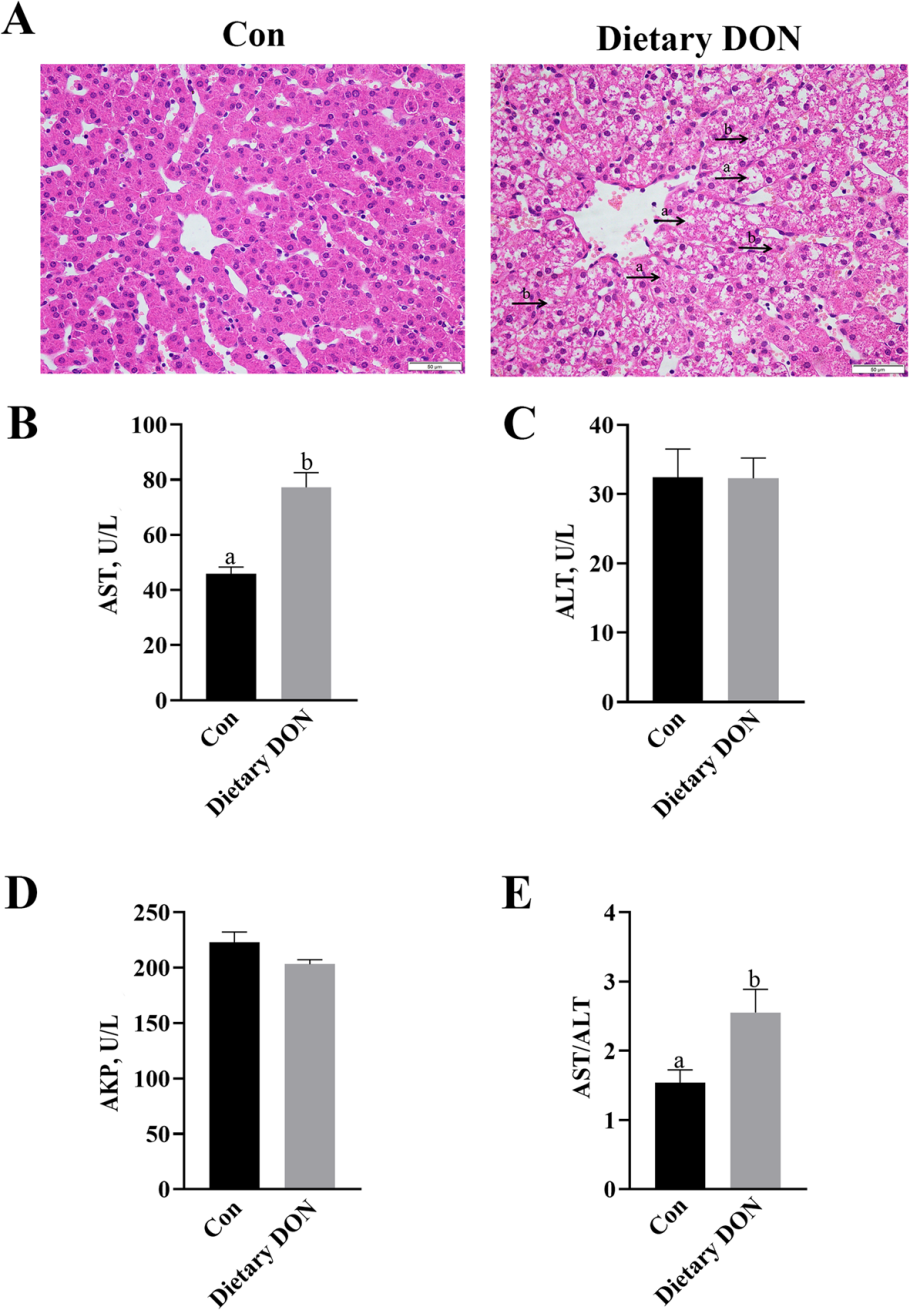
Effects of dietary DON in piglet’s liver morphology and enzymes. Control (Con) group was fed a basal diet, and DON group was fed a diet containing 4 mg/kg DON. **A** Haematoxylin/eosin-stained liver sections. (a) hepatocytes nucleolysis and (b) nuclear pyknosis. The magnification was 40×. **B**–**E** Serum AST, ALT, AKP activities and AST/ALT ratio. Values are means ± SE, *n* = 6. ^ab^Values without a common letter differ significantly (*P* < 0.05). AKP, Alkaline phosphatase; ALT, Alanine aminotransferase; AST, Aspartate transaminase; DON, Deoxynivalenol

**Fig. 2:**
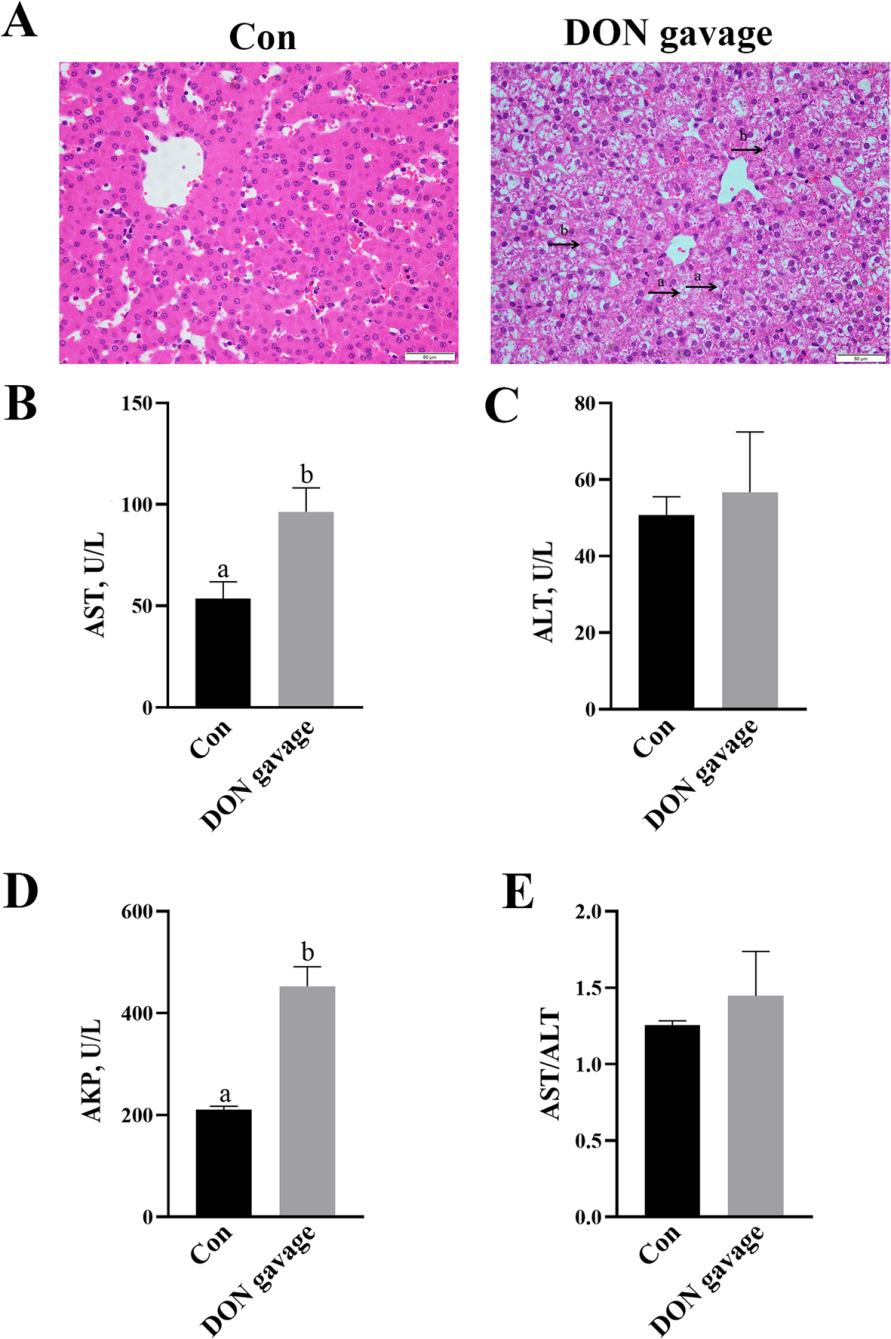
Effects of DON gavage in piglet’s liver morphology and enzymes. Control (Con) group was given a gavage with an equal volume of normal saline, and DON group was given an oral gavage with 2 mg/kg BW DON. **A** Haematoxylin/eosin-stained liver sections. (a) hepatocytes nucleolysis and (b) nuclear pyknosis. The magnification was 40×. **B**–**E** Serum AST, ALT, AKP activities and AST/ALT ratio. Values are means ± SE, *n* = 6. ^a,b^Values without a common letter differ significantly (*P* < 0.05). AKP, Alkaline phosphatase; ALT, Alanine aminotransferase; AST, Aspartate transaminase; DON, Deoxynivalenol

No obvious morphologic changes were found in livers of control pigs (Figs. 1A and 2A). Dietary supplementation or gavage with DON caused nucleolysis, nuclear pyknosis, and disordered arrangement of hepatocyte cords in liver of piglets.

Compared with control group, dietary DON significantly increased serum AST activity and AST/ALT ratio (*P* < 0.05), but had no effect on serum ALT activity (Fig. 1B–E). DON gavage increased DON concentration in serum and liver (Additional file 2). DON gavage significantly increased activities of serum AST and AKP (*P* < 0.05), but had no effect on serum ALT activity and AST/ALT ratio (Fig. 2B–E).

### DON activates liver inflammatory response

**Fig. 3:**
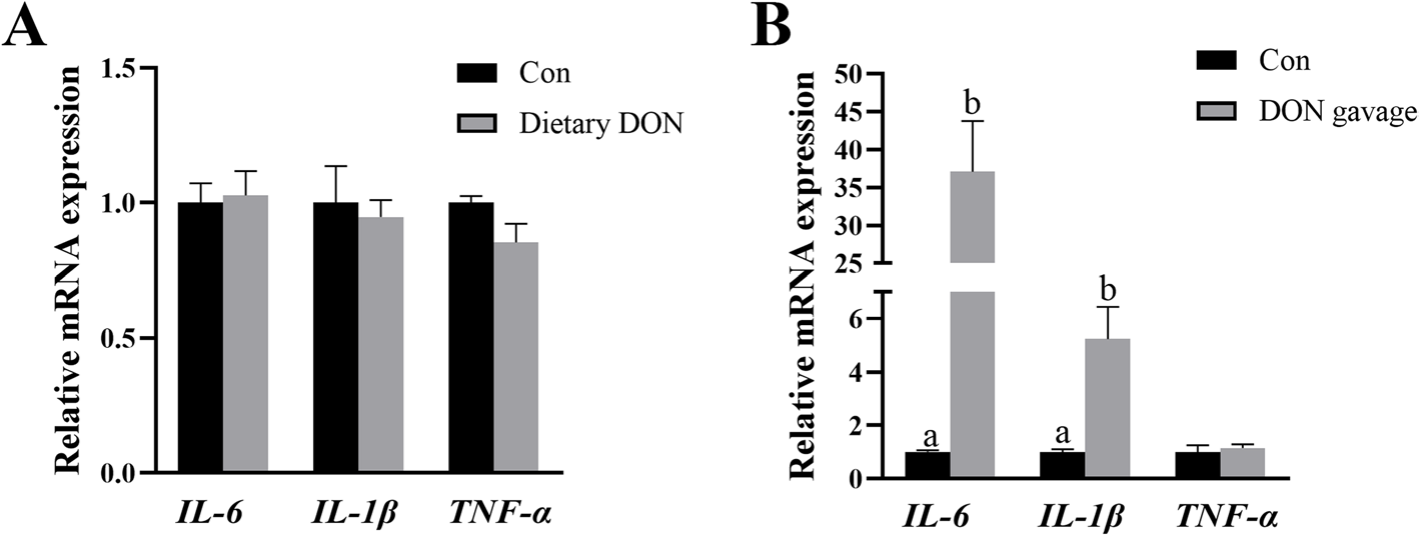
Effects of dietary or gavage with DON on mRNA expression of pro-inflammatory cytokines in liver in piglets. **A** The mRNA expression of *IL-6*, *IL-1β* and *TNF-α* after dietary DON exposure. **B** The mRNA expression of *IL-6*, *IL-1β* and *TNF-α* after DON gavage. Values are means ± SE, *n* = 6. ^a,b^Values without a common letter differ significantly (*P* < 0.05). DON, Deoxynivalenol; *IL-6*, Interleukin-6; *IL-1β*, Interleukin-1β; *TNF-α*, Tumor necrosis factor-α

Compared with control group, dietary DON had no effect on mRNA expression of liver tumor necrosis factor-α (*TNF-α*), interleukin-6 (*IL-6*) and interleukin-1β (*IL-1β*) (Fig. 3A). However, DON gavage significantly increased mRNA expression of *IL-6* and *IL-1β* (*P* < 0.05), but had no effect on mRNA expression of *TNF-α* (Fig. 3B).

### DON induces hepatocyte necroptosis

**Fig. 4:**
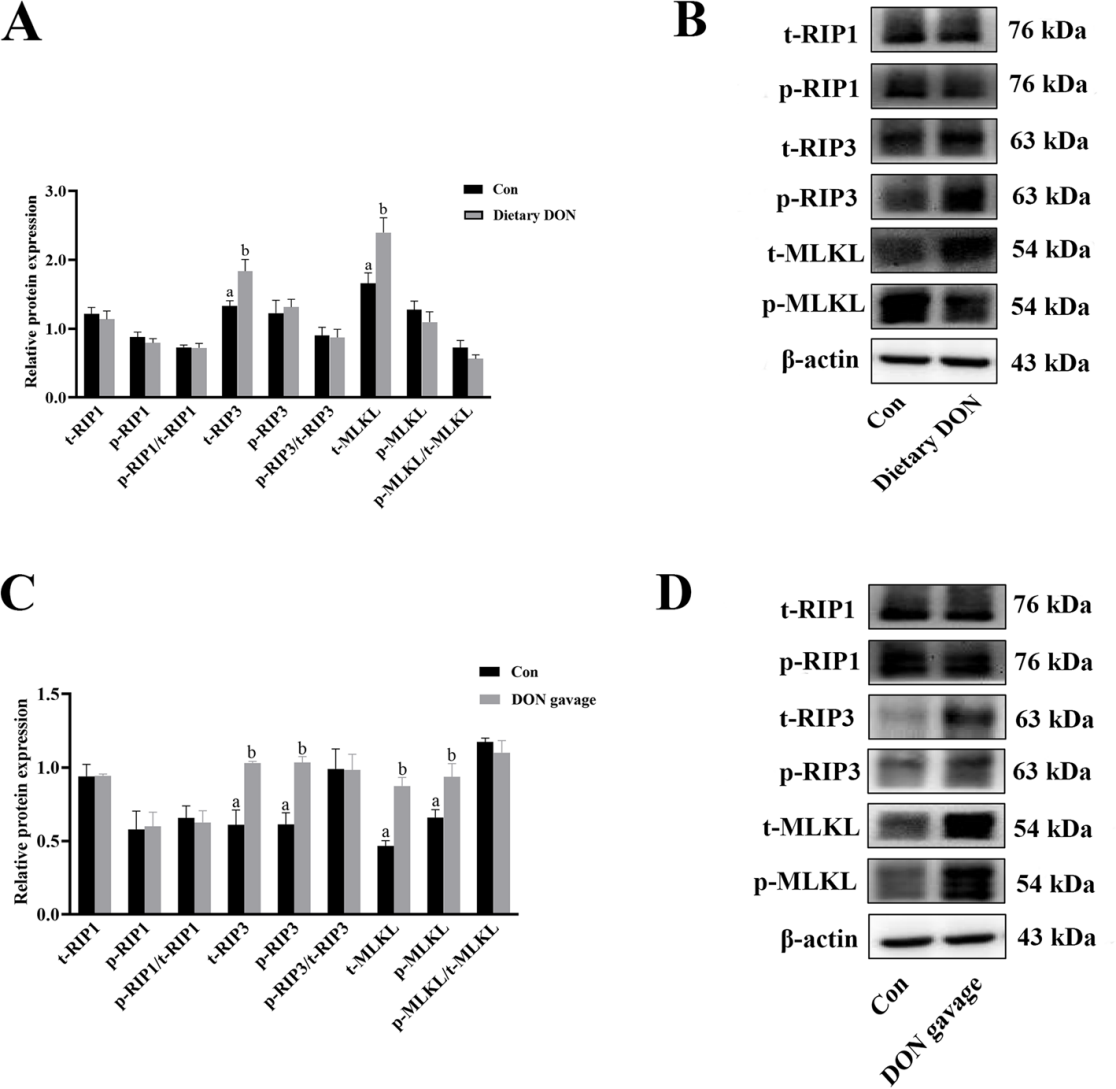
Effects of dietary or gavage with DON on protein expression of necroptosis signals in liver cells of piglets. **A** The protein expression of necroptosis signals after dietary DON exposure. **B** Representative bands of necroptosis signals after dietary DON exposure. **C** The protein expression of necroptosis signals after DON gavage. **D** Representative bands of necroptosis signals after DON gavage. Values are means ± SE, *n* = 6. ^a,b^Values without a common letter differ significantly (*P* < 0.05). DON, Deoxynivalenol; p-MLKL, Phosphorylated mixed lineage kinase domain-like protein; p-RIP1, Phosphorylated receptor interacting protein kinase 1; p-RIP3, Phosphorylated receptor interacting protein kinase 3; t-MLKL, Total mixed lineage kinase domain-like protein; t-RIP1, Total receptor interacting protein kinase 1; t-RIP3, Total receptor interacting protein kinase 3

Compared with control group, dietary DON significantly increased protein abundance of liver t-RIP3 and t-MLKL (*P* < 0.05) (Fig. 4A and B). DON gavage significantly increased protein abundance of liver t-RIP3, p-RIP3, t-MLKL and p-MLKL (*P* < 0.05) (Fig. 4C and D).

### Nec-1 inhibits DON-induced hepatocyte necroptosis

**Fig. 5:**
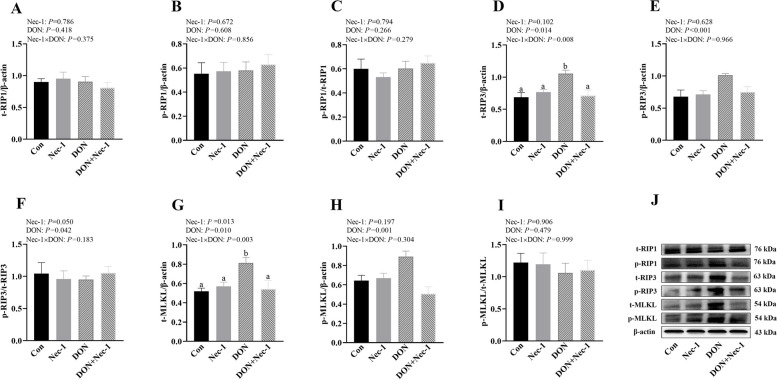
Effects of Nec-1 on protein expression of necroptosis signals in liver of piglets after DON gavage. Piglets were given a gavage with 2 mg/kg BW DON or an equal volume of normal saline after intraperitoneal injection of 0.5 mg/kg BW Nec-1 or an equal volume of 5% dimethylsulfoxide (DMSO). Pigs were euthanized at 6 h after DON or saline gavage. **A–I **Protein expression of necroptosis signals. **J **Representative bands. Values are means ± SE, *n* = 6. ^a,b^Values without a common letter differ significantly (*P* < 0.05). DON, Deoxynivalenol; p-MLKL, Phosphorylated mixed lineage kinase domain-like protein; p-RIP1, Phosphorylated receptor interacting protein kinase 1; p-RIP3, Phosphorylated receptor interacting protein kinase 3; t-MLKL, Total mixed lineage kinase-like protein; t-RIP1, Total receptor interacting protein kinase 1; t-RIP3, Total receptor interacting protein kinase 3

DON gavage increased DON concentration in serum and liver (Additional file 2). However, Nec-1 decreased DON concentration in serum and liver after DON gavage (Additional file 3). In addition, DON gavage significantly increased protein abundance of t-RIP3, p-RIP3, p-RIP3/ t-RIP3, t-MLKL and p-MLKL (*P* < 0.05, Fig. 5). There were Nec-1 × DON interactions (*P* < 0.05) observed for t-RIP3 and t-MLKL, in which Nec-1 decreased the protein expression of t-RIP3 and t-MLKL in DON-challenged piglets, however, t-RIP3 and t-MLKL did not differ among non-DON-challenged piglets (Fig. 5D and J).

### Nec-1 attenuates DON-induced liver injury, dysfunction and inflammation

**Fig. 6:**
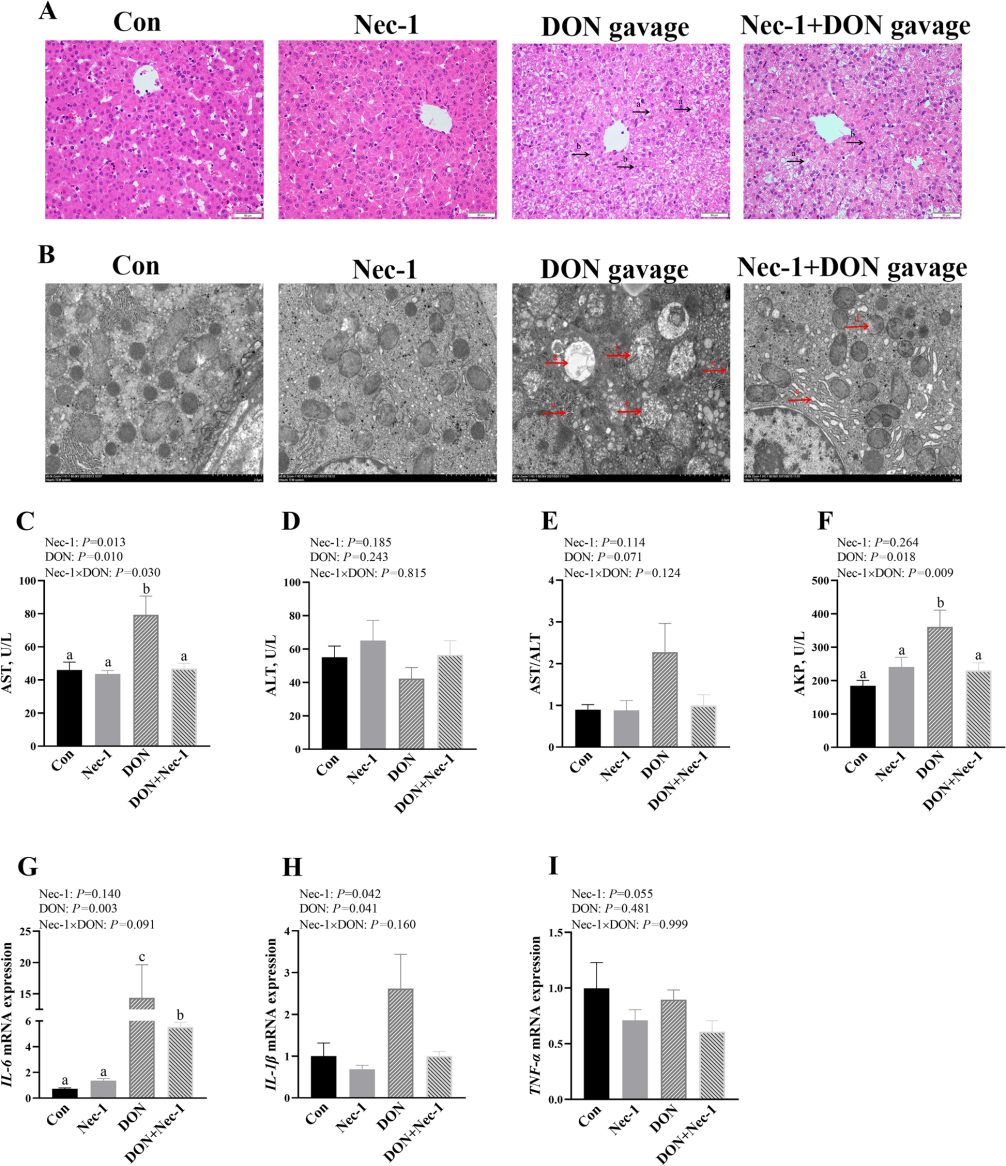
Effects of Nec-1 on liver injury, dysfunction and inflammation in piglets after DON gavage. Piglets were given a gavage with 2 mg/kg BW DON or an equal volume of normal saline after intraperitoneal injection of 0.5 mg/kg BW Nec-1 or an equal volume of 5% dimethylsulfoxide (DMSO). Pigs were euthanized at 6 h after DON or saline gavage. **A** Haematoxylin/eosin-stained liver sections. (a) hepatocytes nucleolysis and (b) nuclear pyknosis. **B** Liver ultrastructure. (a) mitochondrial vacuolation, (b) mitochondrial crest fracture, (c) endoplasmic reticulum dilation and (d) mitochondrial membrane disruption. **C**–**F** Serum AST, ALT, AKP activities and AST/ALT ratio. **G**–**I** The mRNA expression of *IL-6*, *IL-1β* and *TNF-α* in liver of piglets. Values are means ± SE, *n* = 6. ^a–c^Values without a common letter differ significantly (*P* < 0.05). AKP, Alkaline phosphatase; ALT, Alanine aminotransferase; AST, Aspartate transaminase; DON, Deoxynivalenol; IL-6, Interleukin-6; IL-1β, Interleukin-1β; TNF-α, Tumor necrosis factor-α

DON challenge led to liver damage, however, inhibition of necroptosis by Nec-1 pretreatment alleviated DON-caused liver damage indicated by mild hepatocyte nucleolysis, nuclear pyknosis, and disordered arrangement of hepatocyte cords (Fig. 6A). Moreover, Nec-1 alleviated DON-caused mitochondrial vacuolation, mitochondrial crest fracture, and endoplasmic reticulum dilation (Fig. 6B). DON gavage also induced hepatocyte injury with increased cell membrane permeability due to higher AST and AKP activities (Fig. 6C–F). There were Nec-1 × DON interactions observed (*P* < 0.05) for AST and AKP in which Nec-1 decreased the activities of AST and AKP in DON-challenged piglets, whereas the activities of AST and AKP did not differ in non-DON challenged piglets (Fig. 6C–F).

DON gavage significantly increased mRNA expression of *IL-1β* and *IL-6* in liver (*P* < 0.05, Fig. 6G and H). There was a trend for Nec-1 × DON interaction observed (*P* < 0.10) for *IL-6* in which Nec-1 decreased the mRNA expression of *IL-6* in DON-challenged piglets, whereas the mRNA expression of *IL-6* did not differ in non-DON challenged piglets (Fig. 6G–I).

## Discussion

DON is one of the most common and dangerous mycotoxins, which widely exists in feed, posing great threat to animal’s health [2]. In livestock production, DON pollution is inevitable. After DON intake, gastrointestinal tract was the primary target organ. However, as the most important detoxification organ, liver becomes another equally important target organ attacked by DON [8]. However, till now, little is known that how DON damages the liver of piglets.

In present study, we first fed DON-contaminated feed to weaned piglets to investigate the effect of dietary DON on liver. We found that dietary DON led to disorder of hepatocyte cords, liver nuclear lysis, pyknosis, and severe vacuolization of hepatocytes, which suggests that DON caused liver injury. This is consistent with Peng et al. [29] who reported that long-term consumption of DON-contaminated feed led to liver damage in mice. The histological lesions can lead to an increase in cellular permeability of hepatocytes and caused extravasation of enzymes such as AST, ALT and AKP. Therefore, higher AST and ALT activities in plasma indicate hepatocyte damage, and higher AKP activity indicates cholestasis. In our study, the enzymes agreed with the results of changes in cellular morphology of liver. Similarly, Wu et al. [30] also found that long-term feeding of growing pigs with diets containing 3 mg/kg DON significantly increased the activities of serum AST and AKP in pig.

In order to further investigate the effect of DON exposure on liver, we gave an oral gavage with DON to piglets. Expectedly, we found that DON gavage also caused liver morphology damage, which is consistent with the results of DON supplementation in feed. Moreover, DON gavage significantly increased AST and AKP activities in serum of piglets, which suggests that acute DON gavage also led to liver injury. Mikami et al. [31] suggested that injection of DON increased apoptosis of hepatocytes and caused hepatotoxicity. These data showed that both DON chronic feed and acute oral exposures can cause liver injury.

Liver injury is often accompanied by inflammation [32]. In present experiment, we found that DON gavage significantly increased mRNA expression of *IL-6* and *IL-1β* in liver of piglets, which indicated that DON gavage induced liver inflammatory response. Consistently, Barbouche et al. [33] reported that injection of DON for 3 h significantly increased mRNA expression of *IL-6*, *IL-1β* and *TNF-α* in mouse liver. However, dietary DON had no effect on mRNA expression of *TNF-α*, *IL-6* and *IL-1β*. The discrepancies in inflammation might be related to the different concentration and time period of DON exposure to piglets. A longer feeding time might cause liver inflammatory response. For example, Stanek et al. [22] reported that oral gavage of DON (2.4 mg/kg BW/d) for 28 d triggered liver inflammation in mice. Ji et al. [34] found that piglets fed 3 mg/kg DON-contaminated diet for 24 d led to higher concentrations of IL-1β, IL-8 and TNF-α in liver. This was also supported by Zong et al. [35] who reported that pigs fed with DON (4 mg/kg) for 28 d had higher mRNA level of inflammatory genes in liver.

Liver injury is closely related to hepatocyte death [36]. To further investigate if DON-induced liver injury was accompanied by necroptosis. We examined protein expression of necroptosis signals including RIP1, RIP3 and MLKL. We found that dietary DON increased protein expression of t-RIP3 and t-MLKL, and DON gavage also increased protein expression of t-RIP3, p-RIP3, t-MLKL and p-MLKL, which demonstrated that DON exposure activated liver necroptosis signaling pathway. At present, there is no in vivo study exploring the effect of DON on necroptosis signaling pathway in liver. Xiao et al. [37] reported that DON treatment activated necroptosis signaling pathway in IPEC-1 cells. Consistently, Zhou et al. [38] also showed that DON exposure led to intestinal necroptosis in piglets. In our current study, we uncovered for the first time that DON exposure activated liver necroptosis signaling pathway in piglets.

To further demonstrate that DON-induced liver injury was partially due to the contribution of necroptosis, we used Nec-1, a necroptosis inhibitor, which could specifically inhibit the phosphorylation of RIP1 to block signal transduction of necroptosis [20, 39]. We pretreated piglets with Nec-1 half an hour before DON gavage. We found that Nec-1 pretreatment significantly inhibited the increase of t-RIP3, p-RIP3, t-MLKL and p-MLKL protein expression induced by DON challenge. Similarity, Liu et al. [40] also found that Nec-1 pretreatment before LPS challenge significantly reduced protein levels of t-RIP1, t-RIP3, t-MLKL, p-RIP1, p-RIP3 and p-MLKL in intestine of piglets. Moreover, pretreatment with Nec-1 significantly attenuated the increase of AST and AKP activities and AST/ALT ratio in DON-challenged pigs, which indicated that inhibition of necroptosis by Nec-1 attenuated hepatocyte damage after DON challenge. Further TEM analysis demonstrated that Nec-1 alleviated DON-caused mitochondrial vacuolation and mitochondrial crest fracture. In addition, as shown by HE, Nec-1 pretreatment significantly alleviated DON-caused liver morphological injury such as decreased hepatocytes nucleolysis, nuclear pyknosis, and disordered arrangement of hepatocyte cords. Consistently, Majdi et al. [41] reported that inhibition of necroptosis signaling pathway could alleviate liver function damage of mice. These results suggested for the first time that necroptosis contributed to liver injury in piglets.

We finally investigated if necroptosis contributed to liver inflammation. We found that inhibition of necroptosis by Nec-1 downregulated the mRNA expression of *IL-6* and *IL-1β* in liver of piglets. In agreement with our findings, Zhou et al. [42] found that inhibition of necroptosis signaling pathway could alleviate LPS-induced inflammation in hypothalamic–pituitary–adrenal axis of piglets. It was also reported that inhibition of necroptosis could attenuate intestinal inflammation after DON gavage in pigs [38]. These data suggested that the occurrence of necroptosis contributed to liver inflammation after DON exposure in pigs.

## Conclusions

In summary, our results demonstrate for the first time that DON exposure activates necroptosis signaling pathway in liver of piglets, which is accompanied by the impairment of liver morphology and hepatocyte membrane permeability and inflammation. Inhibition of necroptosis by Nec-1 ameliorates DON-induced damage of liver morphology and hepatocyte membrane permeability and inflammation. It is suggested that necroptosis contributes to DON-induced liver injury and inflammation in weaned piglets.

## Supplementary Information

**Additional file 1.** Primers used for real-time PCR analyses.

**Additional file 2.** The concentration of DON in liver and serum of piglets after DON gavage.

**Additional file 3.** Effects on Nec-1 on DON concentration in liver and serum of piglets after DON gavage.

## Data Availability

The data used to support the findings of this study are available from the corresponding author upon reasonable request.

## Abbreviations

AKP: Alkaline phosphatase
ALT: Alanine aminotransferase
AST: Aspartate transaminase
BW: Body weight
Caspase: Cysteine aspartate protease
DMSO: Dimethylsulfoxide
DON: Deoxynivalenol
HE: Hematoxylin-eosin
IL: Interleukin
MLKL: Mixed lineage kinase domain-like protein
Nec-1: Necrostatin-1
p-MLKL: Phosphorylated-mixed lineage kinase domain-like protein
p-RIP1: Phosphorylated-receptor interacting protein kinase 1
p-RIP3: Phosphorylated-RIP3
RIP 1: Receptor interacting protein kinase 1
TEM: Transmission electron microscopy
