# Supplementary material for: Necroptosis contributes to deoxynivalenol-induced liver injury and inflammation in weaned piglets
Source: J Anim Sci Biotechnol. 2024 Dec 3;15:160. doi: 10.1186/s40104-024-01117-1 (PMC11613918; doi:10.1186/s40104-024-01117-1)
Supplement: Supplementary file 1 — Additional file 1. Primers used for real-time PCR analyses. [file 40104_2024_1117_MOESM1_ESM.docx]

**Additional file 1.** Primers used for real-time PCR analyses.

Primer sequence

| Genes | Forward (5'-3'） | Reverse (5’-3’) |
| --- | --- | --- |
| *IL-6* | AAGGTGATGCCACCTCAGAC | TCTGCCAGTACCTCCTTGCT |
| *IL-1β* | GCTAACTACGGTGACAACAATAATG | CTTCTCCACTGCCACGATGA |
| *TNF-α* | AAGACACCATGAGCACTGAGA | CGACCAGGAGGAAGGAGAAG |
| *GAPDH* | CGTCCCTGAGACACGATGGT | GCCTTGACTGTGCCGTGGAAT |
