# Supplementary material for: Necroptosis contributes to deoxynivalenol-induced liver injury and inflammation in weaned piglets
Source: J Anim Sci Biotechnol. 2024 Dec 3;15:160. doi: 10.1186/s40104-024-01117-1 (PMC11613918; doi:10.1186/s40104-024-01117-1)
Supplement: Supplementary file 2 — Additional file 2. The concentration of DON in liver and serum of piglets after DON gavage. [file 40104_2024_1117_MOESM2_ESM.docx]

**Additional file 2** The concentration of DON in liver and serum of piglets after DON gavage

| **Items** | **Con** | **DON gavage** | ***P* value** |
| --- | --- | --- | --- |
| DON concentration in serum | ND^a^ | 0.408±0.071^b^ | 0.015 |
| DON concentration in liver | ND^a^ | 0.505±0.077^b^ | <0.001 |

Piglets were given an oral gavage with 2 mg/kg BW DON or an equal volume of normal saline

^a,b^Means without a common letter differ significantly (*P* < 0.05)

ND: Not detected
