# Supplementary material for: Necroptosis contributes to deoxynivalenol-induced liver injury and inflammation in weaned piglets
Source: J Anim Sci Biotechnol. 2024 Dec 3;15:160. doi: 10.1186/s40104-024-01117-1 (PMC11613918; doi:10.1186/s40104-024-01117-1)
Supplement: Supplementary file 3 — Additional file 3. Effects on Nec-1 on DON concentration in liver and serum of piglets after DON gavage. [file 40104_2024_1117_MOESM3_ESM.docx]

**Additional file 3** Effects on Nec-1 on DON concentration in liver and serum of piglets after DON gavage

| **Items** | **Nec-1** | | **DON** | | **P value** | | |
| --- | --- | --- | --- | --- | --- | --- | --- |
|  | **DMSO** | **Nec-1** | **DMSO** | **Nec-1** | **Nec-1** | **DON** | **Interactions** |
| DON concentration  in serum | ND^a^ | ND^a^ | 0.422±0.065^c^ | 0.321±0.070^b^ | 0.034 | <0.001 | 0.034 |
| DON concentration  in liver | ND^a^ | ND^a^ | 0.483±0.073^c^ | 0.324±0.080^b^ | 0.002 | <0.001 | 0.002 |

Piglets were given a gavage with 2 mg/kg BW DON or an equal volume of normal saline after intraperitoneal injection of 0.5 mg/kg BW Nec-1 or an equal volume of 5% dimethylsulfoxide (DMSO). Pigs were euthanized at 6 h after DON or saline gavage.

^a–c^Means without a common letter differ significantly (*P* < 0.05).

ND: Not detected.
